# HPV Infections among MSM in Shenzhen, China

**DOI:** 10.1371/journal.pone.0096364

**Published:** 2014-05-06

**Authors:** Dong-Yan Zhang, Yue-Ping Yin, Tie-Jian Feng, Fu-Chang Hong, Ning Jiang, Bao-Xi Wang, Xiang-Sheng Chen

**Affiliations:** 1 Chinese Academy of Medical Sciences and Peking Union Medical College Institute of Dermatology, Nanjing, China; 2 National Center for STD Control, China CDC, Nanjing, China; 3 Shenzhen Center for Chronic Disease Control and Prevention, Shenzhen, China; Baylor College of Medicine, United States of America

## Abstract

**Background:**

An increasing incidence of anal cancer among men, especially men who have sex with men (MSM) suggests a need to better understand anal human papillomavirus (HPV) infection among this group.

**Methods:**

A cross-sectional study was conducted among MSM in Shenzhen, China. Blood was collected for HIV serological testing and syphilis serological screening, and anal swabs were collected for HPV genotyping. Difference of HPV prevalence between HIV seropositive and HIV seronegative MSM was assessed by chi-square test. Factors associated with anal canal HPV infection were assessed by univariate and multivariate logistic regression.

**Results:**

A total of 408 MSM were recruited. HIV and HPV prevalence were 6.9% and 36.4%, respectively. HPV was detected in the anal canal in 71.4% of the HIV-positive MSM and in 33.8% of the HIV-negative MSM (P<0.001). Oncogenic types were seen more often in anal specimens of HIV-positive MSM than in specimens of HIV-negative MSM (P = 0.001). The HPV genotypes detected most frequently were HPV06 (8.2%), HPV16 (7.2%), HPV11 (6.4%), HPV18 (4.7%), HPV58 (4.7%), and HPV52 (4.2%).

**Conclusions:**

In this study, HIV positive MSM had a higher burden of HPV infection, especially oncogenic HPV infection. HPV types 52 and 58 were as popular as those types designed for the currently available vaccine (HPV6, 11, 16, 18).

## Introduction

Infection with Human papillomavirus (HPV) is the most common sexually transmitted infection worldwide [1]. More than 40 types of HPV could infect the anogenital region. HPV types that infect the ano-genital location could be divided into oncogenic types and non-oncogenic types [2].

Approximately 85% of anal cancers worldwide are attributed to the oncogenic HPV [3]. Although anal cancers are rare in the general population (1.5 cases/100,000 population)[4], they are more common among men who has sex with men (MSM) and those infected with human immunodeficiency virus (HIV). HIV-positive MSM have an approximately 1.5 times higher prevalence of anal HPV infection and a 5 times higher risk to develop anal cancer than HIV-negative MSM [5]–[7]. The high prevalence of oncogenic HPV infection and its association with anal cancer among MSM call for molecular epidemiological studies on anal HPV infection among MSM [8].

In China, most studies on prevalence of anal HPV infection among MSM were in northern part of China, while the data on molecular epidemiological studies of anal HPV infection in southern China is limited [9]–[12]. The current cross-sectional study was aimed to investigate the HPV prevalence and genotype distribution in a Chinese southern City, Shenzhen.

## Methods

### Ethic statement

The study protocol was reviewed and approved by the Medical Ethics Committee of the Chinese Academy of Medical Sciences Institute of Dermatology in Nanjing. Written informed consent was obtained from each study participant before the interview and testing. Publication of the study data is in accordance with the community standards and approved by the ethics committee.

### Study population and samples

The study was carried out between July and September of 2009 in a sexually transmitted diseases (STD) clinic specially providing health care for MSM in Shenzhen, China. Those eligible to participate were males, at least 18 years old, ever had sex with another man, willing to provide anal swabs and blood for the tests, physically able and willing to provide written informed consent. After an informed consent was obtained, socio-demographic and sexual behavior data were collected through a face-to-face interview by the trained clinical staff in a separate room using a standardized questionnaire. The questions included age, ethnicity, education, marriage status, place of domicile, dwelling status, self-reported sexual orientation, ever had skin lesions in genitalia (including abnormal secretions, bleeding, breakage, and neoplasia in skin or mucous), number of male sex partners in the previous 6 months, lifetime number of male anal sex partners, et al. Blood was collected for serological tests of HIV and syphilis infections, anal swabs were collected for detection of HPV, and urine was collected for detection of Chlamydia and gonococcus infections.

### Sample processing and analysis

Blood samples were collected for the HIV and syphilis serological assessment. The HIV serologic status was determined by enzyme linked immunoassay (Wantai Biological Medicine Company, Beijing, China). And positive tests were confirmed by HIV-1/2 Western blot assay (HIV Blot 2.2 WB; Genelabs Diagnostics, Singapore). The syphilis serologic status was determined by Treponema pallidum-enzyme linked immunoassay (Wantai Biological Medicine Company, Beijing, China). And positive tests were confirmed by tolulized red unheated serum test kit (Rsbio, Shanghai, China)

### HPV detection and genotyping

Anal swabs were collected by trained personnel by rotating a dry nylon flocked swab in the anal canal for about 3 to 4 rotations. All specimens were coded and stored at −70°C before processed further. HPV DNA was extracted by QIAamp DNA Mini Kit (Qiagen, Gaithersburg, MD) according to the manufacturer's instructions. Then HPV DNA was amplified and viral types were determined using the HPV Geno Array test kit (Hybribio, Chaozhou, China) according to the manufacturer's instructions. The GenoArray test is a L1 consensus primer-based PCR assay and is capable of amplifying 21 HPV genotypes including 15 oncogenic HPV types (16, 18, 31, 33, 35, 39, 45, 51, 52,53, 56, 58, 59, 66 and 68) and 6 non-oncogenic HPV types (6, 11, 42, 43, 44, and 81). We considered a specimen to be positive for HPV if it was positive for any of 21 genotypes. We labeled specimen as oncogenic if any of the 15 oncogenic types were detected (16, 18, 31, 33, 35, 39, 45, 51, 52, 53, 56, 58, 59, 66 and 68), regardless of the presence of other genotypes. In contrast, specimens with single or multiple non-oncogenic HPV genotypes (6, 11, 42, 43, 44, and 81) and not co-infection with oncogenic types were classified as non-oncogenic. If a specimen was positive for single or multiple types of HPV6, 11, 16 and 18, it was classified as HPV6/11/16/18 positive.

The operating protocol has been described in detail by previous study [13]. Briefly, PCR was performed with a reaction volume of 25 micro liters (mL) containing 5 mL of DNA template solution, 19.25 mL of the master mixture provided, and 0.75 mL of DNA Taq polymerase solution. The amplicon was denatured subsequently and subjected to hybridization. The test employs a gene chip with a nylon membrane with immobilized type-specific oligonucleotides probes. The final results were detected by colorimetric change on the chip under direct visualization. The positive control and internal control (β-globin) were provided with the kit in each set of PCR to assess the performance of the test. After hybridization, the presence of a positive result for both the positive and the internal control dots within the membrane indicated that the isolated DNA was of good quality, the hybridization kit was valid, and the PCR and hybridization process was performed properly.

### Statistical Analysis

All data from the questionnaires and laboratory tests were double entered and compared using EpiData software (EpiData 3.02 for Windows, The Epi Data Association Odense, Denmark) to ensure the quality of data entry.

Outcome variables include prevalence rates of HPV infections and their 95% confidence intervals (CI). Univariate analysis was used to determine association between socio-demographic, behavioral and biomedical characteristics and the infections, and crude odds ratio (COR) and its 95% CI were calculated. Factors with significance level of p≤0.05 in univariate logistic regression analyses were included in multivariate logistic regression model to explore the association of indicators with acquisition of each of the four HPV outcome variables (any HPV, oncogenic, noncongenic and multiple type infections). Adjusted odds ratio (AOR) and its 95% CI were calculated. Values of p<0.05 were considered statistically significant. Data were analyzed using SPSS (version 18.0 for Windows; SPSS Inc., Chicago, IL).

## Results

### Characteristics of the Participants

The characteristics of the study population (N = 408) were outlined in Table 1. 404 of the anal swabs gave valid genotyping results (4/408 swabs, 0.98%, did not showβ-globin amplification). The majority of MSM were between 20–39 years (88.9%) (data was not shown), Han nationality(95.6%),had an education of high school or less (64.5%), never got married (68.4%), with a domicile outside Guangdong province (Shenzhen is a city of Guangdong) (76.2%). 46.3% of the participants lived alone and only 9.3% lived with their family. Only 32.2% reported themselves as homosexual and 32.9% reported as bisexual. 17.5% ever had skin lesions in genital (including abnormal secretions, bleeding, breakage and neoplasia in skin or mucous, et al). 35.3% reported having less than 2 male anal sex partners, 41.9% reported 2 to 9 male anal sex partners and 22.8% had more than 10 male anal sex partners in the previous 6 months, while that was 37.4%, 38.2% and 24.4% respectively for lifetime male anal sex partners. Laboratory data suggest the prevalence of HIV, syphilis, chlamydia and gonorrhea in the participants are 6.9%, 24.5%, 16.3% and 7.4%, respectively.

**Table 1:** Selected characteristics of the study population.

Characteristic*	
Age, median (IQR), years (n = 408)	29 (25–34.8)
Ethnicity (%) (n = 408)	
Han	95.6
Others	4.4
Education (%) (n = 408)	
High school or less	64.5
University or more	35.5
Marital status (%) (n = 408)	
Never get married	68.4
Married	25.2
Divorced	6.4
Place of domicile (%) (n = 408)	
Shenzhen	9.6
Guangdong (not Shenzhen)	14.2
Other provinces	76.2
Dwelling status (%) (n = 408)	
Live alone	46.3
Live with others	44.4
Live with family	9.3
Self-reported sexual orientation (%) (n = 143)	
Homosexual	32.2
Homosexual, but can have sex with a female	33.6
Bisexual	32.9
Not sure	1.4
Ever had skin lesions in genital (%) (n = 143)	
Yes	17.5
No	82.5
N.male anal sex partners in previous 6 months, median (IQR), (n = 136)	3(2–7)
N.lifetime male anal sex partners, median (IQR), (n = 254)	3(1–8)
Co-infection with other STD (%) (n = 408)	
No	57.4
Yes	42.6
HIV	6.9
Syphilis	24.5
Chlamydia	16.3
Gonorrhea	7.4

*The sum of numbers in some subgroups were less than 408 because some participants did not respond to some questions.

IOR, interquartile range; STD, sexually transmitted diseases.

### Prevalence of anal HPV infection

Prevalence of HPV infection among HIV positive MSM (71.4%) was significantly higher than those MSM negative by HIV (33.8%, P<0.001). Specifically, the difference in prevalence between HIV-positive and HIV-negative MSM was only statistically significant for any types, oncogenic types, multiple types, and single types HPV; however, we did not found that difference for non-oncogenic HPV. HPV6 (8.2%), HPV16 (7.2%), HPV11 (6.4%), HPV18 (4.7%), HPV58 (4.7%), HPV52 (4.2%) were found to be the most frequently identified types. Among oncogenic types, HPV16 and HPV 18 accounted for 40.2% and HPV 58 and HPV 52 are also predominant types of oncogenic HPV.

### Factors related to anal HPV infection

The association between potential risk factors and anal HPV infection was assessed by univariate logistic analyses (Table S1). No significant association between age group and any HPV, oncogenic HPV, multiple HPV infection were found; however, for non-oncogenic HPV, the infection of non-oncogenic HPV decreased in the 30–39 years group compared to the ≥40 years group (COR: 0.31, 95%CI: 0.11–0.89, *p* = 0.028). Ever had skin lesion in genital increased the possibility of any HPV infection (COR: 3.38, 95%CI: 1.37–8.32, *p* = 0.008), oncogenic HPV infection (COR: 2.90, 95%CI: 1.19–7.08, *p* = 0.019) and multiple HPV infection (COR: 3.35 95%CI: 1.09–10.30, *p* = 0.035). Having more than 10 male sexual partners in the recent 6 months facilitated any HPV infection compared to 0–2 male sexual partners (COR:2.59, 95%CI: 1.02–6.61, *p* = 0.046). HIV and syphilis seropositivity were both associated with significantly increased infections by any HPV, oncogenic HPV and multiple HPV, while MSM infected with chlamydia had an increased infection of any HPV, non-oncogenic HPV and multiple HPV. Ethnicity, education, marital status, place of domicile, dwelling status, self-reported sexual orientation, lifetime number of male anal sex partners and co-infection with gonorrhea were not significantly associated with the four HPV infection outcomes.

In multivariate logistic analysis (Table 2), ever had skin lesions in genital was the only factor associated with any HPV infection (AOR: 4.15, 95%CI: 1.50–11.51, *p* = 0.006) and oncogenic HPV infection (AOR: 2.88, 95%CI: 1.16–7.14, *p* = 0.023) after adjusted by confounders and variables remaining in the model. Age was the only factor associated with non-oncogenic HPV infection. Compared with the age group of ≥40 years, those aged 30–39 years had a decreased infection of non-oncogenic HPV (AOR:0.32, 95%CI: 0.11–0.91, *p* = 0.033). No factor was found to be associated with multiple HPV infection in multivariate logistic analysis.

**Table 2:** Factors associated with anal HPV infection among MSM: multivariate logistic analyses.

Factor	Adjusted odds ratio (95% confidence interval)			
	Any HPV	Oncogenic	Nononcogenic	Multiple
Age				
≤19 years	–	–	0.78(0.08–7.96)	–
20–29years	–	–	0.51(0.20–1.31)	–
30–39years	–	–	0.32(0.11–0.91)*	–
≥40years	–	–	Reference	–
Ever had skin lesions in genital				
No	Reference	Reference	–	Reference
Yes	4.15(1.50–11.51)*	2.88 (1.16–7.14)*	–	3.11(0.97–9.98)
Number of anal sex partners in the recent six months				
0–2 men	Reference	–	–	–
3–9 men	1.34 (0.56–3.19)	–	–	–
≥10 men	2.66 (0.99–7.18)	–	–	–
HIV serological status				
Negative	Reference	Reference	–	Reference
Positive	3.55 (0.98–12.84)	2.06 (0.69–6.17)	–	1.54 0.33–7.12)
Syphilis serological status				
Negative	Reference	Reference	–	Reference
Positive	1.12 (0.46–2.73)	0.87 (0.36–2.10)	–	2.43(0.81–7.34)
Chlamydia				
Negative	Reference	–	Reference	Reference
Positive	0.76 (0.28–2.03)	–	2.00 (0.93–4.33)	1.46(0.37–5.73)

*P<0.05 in multivariate logistic regression model.

## Discussion

The prevalence and genotype distribution of HPV varied widely in different places [14]. Since China is vast in territory and HPV prevalence and HPV related health burden are heaviest in MSM [15], it is imperative to survey the baseline HPV genotype distribution in MSM from different parts of the country before vaccine application. Unlike the studies performed in northern China [9]–[12], we examined the prevalence and risk factors for the detection of anal HPV infection in MSM from a southeast city of China in this study. To the best of our knowledge, this is the first molecular epidemiological study on anal HPV infection among MSM from south China.

Our data was similar with a clinic-based study in Rotterdam of the Netherland [16]. The overall prevalence of HIV and anal canal HPV infection was 6.9% and 36.4%, respectively, in our study, and that was 6.6% and 34.9% in the Netherland study. Furthermore, the HPV prevalence in the HIV seropositive group and seronegative group were both similar. However, HPV genotype distribution was different in the two studies, which may be related to different population scales and/or HPV genotyping methods in the studies.

The overall prevalence of HIV and anal canal HPV infection in our study was lower than that from Beijing and Tianjin (8.5% and 62.1%, respectively) [10]. The difference may be related to the recruitment strategies, HPV testing method, or the different study areas. As our study population was recruited from a STD clinic providing healthcare for MSM, it may over-represent MSM who had better access to medical care. Furthermore, the different demographic and behavioral characteristics of the MSM (e.g., older age, higher proportion of bisexual men, fewer male sex partners in lifetime and previous 6 months) in our study from those in Beijing and Tianjin should be considered.

The higher prevalence of any type HPV, oncogenic HPV and multiple HIV in HIV positive MSM than HIV negative MSM was in accordance with several previous studies [5], [6]. Previous studies rarely analyzed the different prevalence of single HPV or non-oncogenic HPV between HIV positive and HIV negative MSM. In the current study, single type HPV infection was found higher in HIV positive MSM than those men negative for HIV, while non-oncogenic HPV infection was not found the difference. HPV6 was the most frequently detected genotype and HPV16 was the most prevalent oncogenic HPV genotype in our study. For the oncogenic HPV genotypes, only prevalence of HPV51 was different in MSM with different HIV serological status, while HPV6 and HPV81 were different for the non-oncogenic HPV genotypes. HPV type 52 and 58 were almost as popular oncogenic genotypes as HPV 18 (4.7%, 4.2% and 4.7%) and the two oncogenic types (HPV16 and HPV18), which were most highlighted in many previous studies [17], were only account for around 40% of all oncogenic types. In a study of HPV prevalence in female sex workers in Guangxi, China, HPV 52 and 58 were also two of most prevalent genotypes [18]. In addition, HPV52 was also found one of the most prevalent genotypes in MSM in Beijing and Tianjin, China [10]. It was known that the currently available quadrivalent HPV vaccine recommended for males [19] was designed for type 6, 11, 16 and 18. However, these vaccines do not cover some oncogenic genotypes frequently reported in the current study, such as HPV 58 and HPV 52.

Previously reported risk factors for anal canal HPV infection in MSM included younger age, minority, larger number of sexual partners a men has currently or has had in his lifetime, having ever had oral sex, and having ever found sex partners in gay venues, recent history of anal warts, and recent receptive anal intercourse [6], [20], [21]. There also was an association between a high prevalence of anal HPV infection and trauma of the anal epithelium, rectal drug use, smoking, HIV infection, hepatitis B infection, positive chlamydia serologic result, and history of gonorrhea [5], [21], [22].

In the current study, the prevalence of any HPV, oncogenic HPV and multiple HPV were all similar across four age groups, just as several previous studies [23]–[26]. However, non-oncogenic HPV infection was supposed to increase after 40 years old. Having ever had skin lesions in genital was associated with higher prevalence of any HPV and oncogenic HPV, but not associated with prevalence of non-oncogenic HPV. The result is similar to that reported in a previous study [6]. It is noted that MSM ever had skin lesions appeared on the genitals have higher possibility of anal oncogenic HPV infections. However, the mechanisms still need to be studied.

In summary, our study confirms a high burden of anal HPV infection among MSM, particularly those MSM positive for HIV in Shenzhen, China. HPV types 52 and 58 are as popular as those types designed for the currently available quadrivalent vaccine (HPV6/11/16/18). The high prevalence of oncogenic HPV infection among MSM calls further for the importance to consider HPV vaccination for protection against anal cancer in this population. Innovative formats of vaccines to cover more oncogenic types of HPV may be needed to designed and developed for the population in China.

## Supporting Information

Table S1 **Factors associated with anal HPV infection among MSM: univariate logistic analyses.**
(DOC)
