# Supplementary material for: HPV Infections among MSM in Shenzhen, China
Source: PLoS One. 2014 May 6;9(5):e96364. doi: 10.1371/journal.pone.0096364 (PMC4011743; doi:10.1371/journal.pone.0096364)
Supplement: Table S1 — Factors associated with anal HPV infection among MSM: univariate logistic analyses. (DOC) [file pone.0096364.s001.doc]

Table S1. Factors associated with anal HPV infection among MSM: univariate logistic analyses

| Factor | Crude Odds ratio (95% confidence interval) | | | |
| --- | --- | --- | --- | --- |
|  | Any HPV | Oncogenic | Non-oncogenic | Multiple types |
| Age |  |  |  |  |
| ≤19 years | 1.60(0.29-8.98) | 1.94(0.30-12.53) | 0.91(0.09-9.10) | 2.40(0.21-27.78) |
| 20-29years | 1.04(0.52-2.10) | 1.55(0.67-3.57) | 0.56(0.22-1.41) | 1.53(0.44-5.38) |
| 30-39years | 0.73(0.35-1.52) | 1.29(0.55-3.05) | 0.31(0.11-0.89) | 1.47(0.41-5.28) |
| ≥40years | Reference | Reference | Reference | Reference |
| Ethnicity |  |  |  |  |
| Han | Reference | Reference | Reference | Reference |
| Others | 1.12(0.42-2.95) | 0.79(0.25-2.44) | 1.89(0.52-1.82) | 1.68(0.47-6.06) |
| Education |  |  |  |  |
| High school or less | Reference | Reference | Reference | Reference |
| University or more | 0.75(0.49-1.16) | 0.80(0.50-1.29) | 0.76(0.38-1.55) | 0.50(0.24-1.05) |
| Marital status |  |  |  |  |
| Never get married | Reference | Reference | Reference | Reference |
| Married | 1.02(0.64-1.64) | 1.16(0.70-1.90) | 0.74(0.33-1.69) | 0.91(0.44-1.88) |
| Divorced | 0.93(0.40-2.16) | 0.67(0.24-1.85) | 1.60(0.52-4.99) | 0.30(0.04-2.32) |
| Place of domicile |  |  |  |  |
| Shenzhen City | Reference | Reference | Reference | Reference |
| Other area in Guangdong | 2.19(0.88-5.45) | 2.48(0.88-6.95) | 1.13(0.25-5.04) | 2.50(0.64-9.75) |
| Other provinces | 2.00(0.92-4.36) | 2.04(0.82-5.04) | 1.40(0.41-4.80) | 1.35(0.39-4.63) |
| Dwelling status |  |  |  |  |
| Live alone | Reference | Reference | Reference | Reference |
| Live with others | 0.82(0.54-1.25) | 0.83(0.52-1.32) | 0.91(0.47-1.76) | 1.54(0.78-3.03) |
| Live with family | 0.54(0.25-1.18) | 0.78(0.35-1.75) | 0.22(0.03-1.66) | 2.03(0.74-5.57) |
| Self-reported sexual orientation |  |  |  |  |
| Homosexual | Reference | Reference | Reference | Reference |
| Homosexual but occasional sex with a female | 0.67(0.28-1.62) | 0.89(0.35-2.29) | 0.37(0.07-2.03) | 1.23(0.35-4.36) |
| Bisexual | 1.94(0.85-4.44) | 1.46(0.60-3.57) | 1.94(0.60-6.31) | 0.76(0.19-3.04) |
| Not sure | 1.71(0.10-29.07) | 2.83(0.16-48.93) | 0 | 8.20(0.44-152.52) |
| Ever had skin lesions in genital |  |  |  |  |
| No | Reference | Reference | Reference | Reference |
| Yes | 3.38(1.37-8.32)* | 2.90(1.19-7.08)* | 1.65(0.49-5.62) | 3.35(1.09-10.30)* |
| N. male anal sex partners in the recent six months |  |  |  |  |
| 0-2 men | Reference | Reference | Reference | Reference |
| 3-9 men | 1.38(0.61-3.12) | 1.90(0.78-4.63) | 0.48(0.11-2.10) | 1.54(0.42-5.61) |
| ≥10 men | 2.59(1.02-6.61)* | 1.51(0.53-4.30) | 2.92(0.86-9.97) | 1.59(0.37-6.91) |
| N. lifetime male anal sex partners |  |  |  |  |
| 0-2 men | Reference | Reference | Reference | Reference |
| 3-9 men | 0.78(0.43-1.42) | 0.58(0.30-1.15) | 1.55(0.61-4.00) | 0.77(0.29-2.05) |
| ≥10 men | 1.19(0.62-2.30) | 1.32(0.66-2.64) | 0.76(0.22-2.65) | 1.67(0.65-4.28) |
| HIV serological status |  |  |  |  |
| Negative | Reference | Reference | Reference | Reference |
| Positive | 4.90(2.10-11.44)* | 3.56(1.63-7.76)* | 2.12(0.76-5.92) | 3.05(1.22-7.67)* |
| Syphilis serological status |  |  |  |  |
| Negative | Reference | Reference | Reference | Reference |
| Positive | 1.66(1.05-2.63)* | 1.66(1.02-2.72)* | 1.19(0.57-2.48) | 2.14(1.11-4.11)* |
| Chlamydia |  |  |  |  |
| Negative | Reference | Reference | Reference | Reference |
| Positive | 1.83(1.07-3.11)* | 1.37(0.77-2.43) | 2.13(1.01-4.52)* | 2.13(1.03-4.38)* |
| Gonorrhea |  |  |  |  |
| Negative | Reference | Reference | Reference | Reference |
| Positive | 1.83(0.87-3.87) | 1.96(0.91-4.21) | 1.01(0.29-3.50) | 1.72(0.62-4.74) |

* P<0.05 in univariate logistic regression model.
